# Delaying Onset of Dementia: Are Two Languages Enough?

**DOI:** 10.1155/2014/808137

**Published:** 2014-05-18

**Authors:** Morris Freedman, Suvarna Alladi, Howard Chertkow, Ellen Bialystok, Fergus I. M. Craik, Natalie A. Phillips, Vasanta Duggirala, Surampudi Bapi Raju, Thomas H. Bak

**Affiliations:** ^1^Department of Medicine, Division of Neurology, Baycrest, Toronto, ON, Canada M6A 2E1; ^2^Department of Medicine, Division of Neurology, Mt. Sinai Hospital, Toronto, ON, Canada M5G 1X5; ^3^Rotman Research Institute, Baycrest, 3560 Bathurst Street, Toronto, ON, Canada M6A 2E1; ^4^Department of Medicine (Neurology), University of Toronto, Toronto, Canada M5G 2C4; ^5^Department of Neurology, Nizam's Institute of Medical Sciences, Punjagutta, Hyderabad 500 082, India; ^6^Bloomfield Centre for Research in Aging, Lady Davis Institute for Medical Research and Jewish General Hospital/McGill University Memory Clinic, Jewish General Hospital, 3744 Chemin de la Côte-Sainte-Catherine, Montreal, QC, Canada H3T 1E2; ^7^Division of Geriatric Medicine, Department of Medicine, McGill University, Canada H3T 1E2; ^8^Department of Clinical Neurosciences, Sir Mortimer B. Davis—Jewish General Hospital, Canada H3T 1E2; ^9^Department of Psychology, York University, 4700 Keele Street, Toronto, ON, Canada M3J IP3; ^10^Department of Psychology, University of Toronto, Toronto, ON, Canada M5S 3G3; ^11^Department of Psychology/Centre for Research in Human Development, Concordia University, 7141 Sherbrooke Street West, Montreal, QC, Canada H4B 1R6; ^12^Centre for Research on Brain, Language, and Music, McGill University, Montreal, Canada H3G 2A8; ^13^Department of Linguistics, University College of Arts & Social Sciences, Osmania University, Hyderabad 500 007, India; ^14^Centre for Neural and Cognitive Sciences, University of Hyderabad, Gachibowli, Hyderabad 500 046, India; ^15^Human Cognitive Neuroscience and Centre for Cognitive Aging and Cognitive Epidemiology School of Philosophy, Psychology and Language Sciences, University of Edinburgh, 7 George Square, Edinburgh EH8 9JZ, UK; ^16^Centere for Clinical Brain Sciences, University of Edinburgh, 49 Little France Crescent, Edinburgh EH16 4SB, UK

## Abstract

There is an emerging literature suggesting that speaking two or more languages may significantly delay the onset of dementia. Although the mechanisms are unknown, it has been suggested that these may involve cognitive reserve, a concept that has been associated with factors such as higher levels of education, occupational status, social networks, and physical exercise. In the case of bilingualism, cognitive reserve may involve reorganization and strengthening of neural networks that enhance executive control. We review evidence for protective effects of bilingualism from a multicultural perspective involving studies in Toronto and Montreal, Canada, and Hyderabad, India. Reports from Toronto and Hyderabad showed a significant effect of speaking two or more languages in delaying onset of Alzheimer's disease by up to 5 years, whereas the Montreal study showed a significant protective effect of speaking at least four languages and a protective effect of speaking at least two languages in immigrants. Although there were differences in results across studies, a common theme was the significant effect of language use history as one of the factors in determining the onset of Alzheimer's disease. Moreover, the Hyderabad study extended the findings to frontotemporal dementia and vascular dementia.

## 1. Introduction

Recent studies from Canada [1, 2] and India [3] suggest that speaking two or more languages may significantly delay onset of symptoms of dementia by up to 5 years. However, other results show that an overall protective effect requires proficiency in at least 3 to 4 languages, whereas speaking two languages may delay onset of dementia only in immigrants [4].

Although the mechanisms underlying possible protective effects of bilingualism on dementia onset are unknown, these may relate to cognitive reserve [5], a concept suggesting that complex mental activity can result in brain changes that compensate for cognitive decline due to normal aging or even brain damage [6–8]. Cognitive reserve has been associated with factors such as higher levels of education [9, 10], occupational status [9], social networks [11], and physical exercise [12–14].

In the case of bilingualism, cognitive reserve may arise from reorganization and strengthening of neural networks due to enhanced executive control. There is substantial evidence that language processing in bilinguals involves ongoing activation of both spoken languages. Thus, there is a need for management of attention to the two activated languages, continuous monitoring to determine which language is appropriate, and rapid switching between languages in response to changes in the environment [5]. Since these processes involve executive control, bilinguals are essentially “exercising” their executive systems almost constantly. This may contribute significantly to mechanisms underlying the development of cognitive reserve in bilinguals, although there may be individual differences impacting upon these mechanisms.

Evidence for enhanced executive control in bilinguals comes from studies in children and adults (see Bialystok et al. [5] for review). In addition, studies in preverbal infants showed that those raised in bilingual homes demonstrated greater perceptual attentiveness to facial cues associated with speech production in different languages compared to children raised in monolingual homes [15], as well as enhanced domain-general components of executive function [16]. This suggests that neural changes related to the bilingual experience may start as early as the first year of life prior to the development of speech. This paper reviews evidence for the protective effects of bilingualism on dementia with a focus on a multicultural perspective.

## 2. Canadian Studies

### 2.1. Toronto

The initial study designed to test the concept that bilingualism may delay onset of dementia was carried out at Baycrest Centre for Geriatric Care in Toronto, Canada [1]. This was followed by a second confirmatory report [2]. The first study included patients with dementia due to different causes [1], whereas the second focused only on Alzheimer's disease (AD) [2]. The criterion for bilingualism was that patients spent the majority of their lives, at least from early adulthood, regularly using at least two languages. In both studies the main outcome measure was age of onset of cognitive symptoms. This information was obtained while taking the case history at the initial clinic visit. Although determining when symptoms first appear involves a subjective estimate, there was no reason for a systematic bias in obtaining this data from monolinguals as compared to bilinguals. Age at diagnosis was not used since this is associated with variability due to several factors including when a person decides to seek medical care, wait times for physician visits and investigations, and physician comfort in making a diagnosis, especially in early cases. All patients were diagnosed by consensus involving at least two physicians (neurologist, geriatrician, or geriatric psychiatrist) and a neuropsychologist.

In Study 1, two hundred twenty-eight consecutive charts of patients from the Sam and Ida Ross Memory Clinic were reviewed [1]. Forty-four patients were excluded: 23 because they did not have dementia and 21 who could not be classified as bilingual or monolingual. The final sample consisted of 184 patients. One hundred thirty-two had probable AD and 52 had dementia due to other causes comprised of possible AD, cerebrovascular disease, and non-AD degenerative dementia. Study 2 involved 211 consecutive patients from the Sam and Ida Ross Memory Clinic with probable AD [2]. There was no overlap in patients between the two studies.

There were speakers of 25 different first languages in Study 1 and 21 different languages in Study 2. The most common were Polish, Yiddish, Hungarian, German, and Romanian (Study 1) and Polish, Yiddish, Hungarian, Italian, and French (Study 2).

Both studies showed a significant delay in onset of symptoms of dementia in bilinguals (Table 1). The initial study involving the mixed group of cases with dementia showed a delay of 4.1 years. Separating AD from the other dementias showed a significant delay of 4.3 years in AD and 3.5 years in the other dementias. The study involving only patients with AD showed a delay of 5.1 years. Neither education nor occupational status could account for the findings. Importantly, bilinguals had significantly less education than monolinguals in both studies. Moreover, occupational status did not differ significantly between language groups in Study 1 and was higher in monolinguals in Study 2. This rules out the possibility that the findings favouring bilinguals could be due to these factors. In addition, there were no effects of gender.

The proportion of immigrants differed between bilinguals and monolinguals in both studies. Whereas most bilinguals were immigrants, the minority of monolinguals were immigrants. However, immigration status does not appear to account for the results. In Study 1, analysis of immigrants separately showed a significant delay of 11.5 years in onset of dementia in bilinguals compared to monolinguals. In Study 2, controlling for immigration status in the analysis did not change the results.

There was no difference in severity of dementia between monolinguals and bilinguals at initial visit based on MMSE scores. Moreover, rate of cognitive decline was assessed in a subgroup of patients in Study 1 who had follow-up MMSE scores over 4 years, that is, 24 bilinguals and 25 monolinguals, and these rates were also equivalent for monolingual and bilingual patients. Finally, there was no suggestion that the delay in onset of dementia in bilinguals was an artefact of their waiting longer than monolinguals before consulting a physician. In Study 2, the time interval between first symptoms and first clinic visit was significantly shorter in bilinguals compared to monolinguals. In Study 1, the interval was also shorter for bilinguals although the difference was only marginally significant (*P* = 0.06).

In addition to the above studies suggesting that bilingualism delays the onset of dementia by up to five years, there are two Toronto neuroimaging studies supporting a protective effect of bilingualism on brain function. Schweizer et al. [17] studied monolingual and bilingual patients with AD who were matched on cognitive performance and education and found significantly greater medial temporal lobe atrophy in bilinguals using linear measurements derived from CT scans. There were no differences in atrophy in other areas. These findings are in keeping with the hypothesis that bilinguals have greater cognitive reserve than monolinguals since they have comparable cognitive function to monolinguals despite showing greater medial temporal lobe atrophy. Additionally, Luk et al. [18] found that healthy elderly bilinguals showed better maintained white matter connectivity between frontal and posterior brain regions than did their monolingual counterparts; the authors suggest that this may be a marker for higher levels of brain reserve in bilinguals.

### 2.2. Montreal

Chertkow et al. [4] took advantage of the bilingual French and English nature of Montreal to attempt to replicate the Toronto findings. They examined the database of the Jewish General Hospital Memory Clinic which contained information on 1,842 individuals. The sample was restricted to individuals with memory complaints who were diagnosed with probable AD (*n* = 632). Diagnosis was made by a neurologist or geriatrician in consultation with other Memory Clinic physicians, nurses, and neuropsychologists using NINCDS-ADRDA criteria [19].

Age at diagnosis was available in all cases and served as the main outcome measure (Table 2). This contrasts with age of cognitive symptom onset as the main outcome measure in the Toronto and Indian studies. Diagnosis was usually made at the first visit to the Memory Clinic. Initial diagnosis was mild cognitive impairment (MCI) for 130 subjects. These patients were seen annually in followup. The date of onset of dementia was defined as the clinic visit at which the diagnosis was changed from MCI to AD. In a subset of dementia cases, followup occurred a year after diagnosis was made.

Three levels of language ability were defined: monolingual, bilingual, and multilingual. Multilingualism was defined differently based on the language groups being compared. For comparison of those speaking one versus multiple languages, multilingualism was defined as speaking two or more languages. For analyses of effects of increasing numbers of languages, bilingualism was defined as speaking two languages, whereas multilingualism was defined as speaking three or more languages. When examining bilingualism in the nonimmigrant population, only those who spoke both French and English since youth were considered. The monolingual cohort was made up of only English or French speakers.

There was no direct information of immigrant/native status. Therefore, immigrants were defined as individuals whose first language was not native to Canada, that is, neither English nor French. In contrast, individuals whose first language was English or French were considered native-born. Bilinguals and multilinguals were defined according to the criterion set out by Bialystok and colleagues for bilingualism, that is, those who spent the majority of their lives, at least from early adulthood, regularly using at least two languages [1, 2].

There were 253 multilinguals and 379 monolinguals. Twenty-five first languages were spoken. The most common were English, Polish, French, Yiddish, and Hungarian.

#### 2.2.1. Overall Impact of Bi- or Multilingualism on Age of Diagnosis of AD

There was no significant effect of language status (monolingual versus multilingual) overall on age of dementia diagnosis. However, there was an impact of number of languages spoken when assessed by regression analysis. Considering all language groups, there was a significant positive relation between number of languages spoken and delay in diagnosis of dementia. Education or sex did not account for the findings. Analysis of number of languages spoken showed that those who spoke four or more were diagnosed at a significantly older age than those who spoke one or two languages. There was also a trend for those who spoke three languages to be diagnosed later than those who spoke one or two languages. There was no difference between those who spoke one or two languages. These results suggest that speaking four or more languages is protective, speaking three languages has marginal benefit, and speaking two languages has no benefit. In contrast to the protective effect of speaking more than two languages, being an immigrant led to earlier onset of dementia and thus had a negative impact. However, within the immigrant group, bilingualism did in fact have a protective effect in delaying onset of dementia.

#### 2.2.2. Impact of Bilingualism in Nonimmigrant Cohort

One motivation for the study was to examine the effect of bilingualism without the potential confound of differences in cultural and life experience between individuals born in Canada and immigrants. Thus an additional analysis that was restricted to Canadian born subjects was carried out in English and French monolinguals compared with French/English bilinguals. There were 356 monolinguals (290 English-speaking, 66 French-speaking) and 43 bilinguals (19 with English as first language and 24 with French as first language). Therefore, the monolinguals were 81% English speaking compared with only 19% French speaking, and in the whole sample, there were 89% monolinguals and 11% bilinguals, so the results must be interpreted with caution. In this group, onset of dementia was significantly earlier in bilinguals as compared to monolinguals. Thus being bilingual appeared to have an adverse effect on age of diagnosis in individuals born in Canada.

Further analysis showed that among monolinguals, French speakers were diagnosed 5.3 years earlier than English speakers, while in bilinguals there was no difference between language groups. In the larger native English and French group, including speakers of more than two languages and bilinguals who spoke additional languages other than English or French, there was no difference in age of diagnosis for the native English group based on number of languages spoken when controlling for education and gender, while in the native French group, there was a trend towards significance (*P* = 0.08). Looking only at bilinguals versus monolinguals in the native French group, there was a 3.2-year difference with bilinguals being older at diagnosis. However, the difference was not significant. This suggests that, in the native English group, the number of languages spoken did not provide or contribute to a later diagnosis, while in the native French population, it trended in that direction, though again most strongly for persons speaking more than two languages.

#### 2.2.3. Impact of Number of Languages Spoken in Immigrant Subgroup

The immigrant group was examined separately to see if there was a similar pattern compared to the nonimmigrant group. Monolinguals were diagnosed 5 years earlier than bilinguals, 6.4 years earlier than trilinguals, and 9.5 years earlier than those speaking four or more languages. Also, there was a significant difference between multilingual speakers of four or more languages and bilinguals, with multilinguals being diagnosed 4.5 years later on average. In the immigrant subgroup, there was an impact of number of languages spoken, both at the level of bilingualism and at the level of four or more languages. However, monolinguals had significantly less education than all other language groups, a factor that could have contributed to earlier onset of dementia.

#### 2.2.4. Impact of Cultural Group (Native English, Native French, Immigrant)

To examine the patterns uncovered by separating the cohort into Canadian born subjects whose first language was English, Canadian born subjects whose first language was French, and immigrants, the difference in age of diagnosis between these groups within each linguistic group was analyzed. In the monolingual group, there was a significant difference in age of diagnosis. Canadian born subjects whose first language was English were diagnosed significantly later than Canadian born subjects whose first language was French (5.4 years later) and immigrants (6.6 years later). There was no significant difference between Canadian born subjects whose first language was French and immigrants. In the bilingual and multilingual (three or more languages) groups, there was no significant difference between the three groups. This suggests that native English speaking monolinguals are diagnosed later than either French-speaking native Canadians or immigrant monolinguals. However, this difference disappears in bilinguals and multilinguals. One interpretation of this was that there was a protective effect of multiple languages which emerged much more strikingly in the French-speaking and immigrant populations, but that monolingual English speaking individuals were already “protected” in some way. An alternative explanation is that lack of significance was due to small sample size which was 24 in native Canadians whose first language was French and who spoke two languages and four in those who spoke three languages. None spoke more than three languages. Only 27 native Canadians whose first language was English spoke more than two languages. Fifty-four immigrants spoke more than two languages.

#### 2.2.5. Impact of Occupation

Higher occupation status and more intellectually stimulating work are associated with retained cognitive function in old age [20, 21] and reduced effects of dementia [22]. Given the difference in age of diagnosis between native English monolinguals and the other monolinguals, the possible impact of occupation in protecting against onset of dementia in the monolinguals was examined. There were data on occupational status for 322/379 monolinguals. Native French monolinguals had higher occupation status than either native English or immigrant monolinguals who were not different from each other. This shows that the difference seen in the age of diagnosis between native English monolinguals and the other monolinguals is not attributable to occupational status.

### 2.3. Indian Study

India offers an appropriate environment to study the association between bilingualism and age of dementia onset due to its high degree of linguistic diversity [23] and consequently strong background of bilingualism and multilingualism [24]. In most parts of India, people are exposed to various languages that serve communicative functions differently in different domains such as home, school, and workplace. In some cases, such as intercommunity marriages, children are exposed to two languages right from birth, and in other cases the exposure occurs later after the first language is fully developed. For example, education policies in many states require compulsory instruction in English and Hindi (national official languages) as well as another regional language in schools. Bilingualism and multilingualism may also arise in late adulthood due to migration [25]. In other words, the bilingual and multilingual environment in India is contact based and results largely from intercultural, intergroup interactions involving both literates and semiliterates or even illiterates. For a larger discussion of the role of historical, political, and cultural contextual factors in shaping the multilingual character of India, see Vasanta [26]. With increasing interest in the potentially protective effect of bilingualism on age of onset of dementia [1, 2, 4], a study was undertaken to investigate this association in a memory clinic in Hyderabad, a city in South India [3].

Several epidemiological studies from India demonstrate a high burden of dementia and cognitive disorders [27]. It is estimated that by 2025, around 75% of people aged over 60 years will reside in developing countries, and the number of people living with dementia will double over the next twenty years. This rapid increase has been attributed to the phenomenon of demographic transition, which has resulted in increased life expectancy, as well as urbanization and lifestyle factors [28, 29]. The increasing prevalence of dementia has resulted in development of specialist services for dementia in a few centres across India, including Hyderabad. The Hyderabad Memory Clinic offers comprehensive care that includes diagnosis and multidisciplinary treatment. Patients are evaluated using standard and locally validated neuropsychological tests in addition to imaging studies. In the patient population who visit this memory clinic, AD is the most common subtype of dementia, followed by a relatively high proportion of vascular dementia (VaD) cases. The latter is a reflection of the high cardiovascular disease burden in India. Other dementia subtypes such as frontotemporal dementia (FTD) and dementia with Lewy bodies (DLB) are also frequent [30]. Furthermore, a wide range of educational backgrounds and high degree of multilingualism characterise this cohort of dementia patients.

In Hyderabad, Telugu is spoken by the majority group who are primarily Hindus, whereas the language of a minority group of Muslims is Dakkhini Urdu, a variety of Hindi spoken in the Deccan plateau that includes Hyderabad. As in other parts of India, in the state of Andhra Pradesh (for which Hyderabad is the capital) English is gradually acquiring more and more functional roles in education, administration, and mass media. In addition, Hindi is spoken as the official national language and is taught as a subject at school level. Thus, most people in Hyderabad are exposed to Telugu and Urdu in informal contexts and Hindi and English in formal contexts. A study exploring patterns of multilingualism in Hyderabad suggested that no single language catered to the needs of both Telugu and Urdu speakers' day-to-day life in Hyderabad. A language-use history questionnaire study on Telugu and Dakkhini Urdu speakers indicated that while the former group manage most of their communication needs using Telugu and English (and are functionally bilingual) the latter group are trilingual in that they tend to use Dakkhini Urdu, Telugu, and English in different situations and for different purposes [31]. This widespread use of multiple languages in Hyderabad offered a unique opportunity to explore the association between multilingualism and age of onset of dementia.

In the Hyderabad Memory Clinic study, case records of 648 consecutive patients in the dementia registry were reviewed for age, gender, age of onset of dementia, education, and age when the diagnosis was made [3]. Age of onset of dementia was defined as the age at which the first clinical symptom suggestive of dementia was observed by family members. Educational status was derived from years of formal education received. Language history was obtained from a reliable family member by recording the number of languages spoken fluently by the patient before onset of dementia. Bilinguals were defined as persons with an ability to meet their normal communicative demands in two or more languages when interacting with speakers of any or all of these languages [32]. In addition, these individuals spent the majority of their lives, from late childhood and early adulthood, regularly using at least two or more languages [33].

Four-hundred twenty-four of the 648 consecutive dementia cases were men (65.4%). The mean age of the group at presentation was 66.2 years (range 32–92) and duration of illness ranged from 6 months to 11 years (mean 2.3; SD 1.8). AD was diagnosed in 240 (37.0%), VaD in 189 (29.2%), FTD in 116 (17.9%), DLB in 55 (8.5%), and mixed AD with cerebrovascular disease in 48 (7.4%). Three hundred ninety-one cases (60.3%) were bilingual [3].

The age of onset of dementia among bilinguals was 4.5 years later than in monolinguals [3]. Mean age of onset of dementia among monolinguals was 61.1 years and bilinguals 65.6 years. This difference was significant. A statistically significant delay in age of onset was found in AD (3.2 years), FTD (6.0 years), and VaD (3.7 years) (Table 3). Educational heterogeneity in the Indian cohort made it possible to study the potential confounding effect of education on age of onset. Bilinguals had a significantly higher educational status (12.9 years) and were more often men (74.9%) compared to monolinguals (5.9 years of education, 51% men). Bilingualism had a significant effect on age of onset of dementia (*P* = 0.027) after adjusting for other variables [3]. Further, the absence of immigrants in the Indian cohort removed any possible confounding effect of immigration described in previous studies [1, 2, 4]. Therefore, in the Hyderabad cohort of dementia patients, bilingualism was associated with a significant delay in age of onset of dementia. This association could not be attributed to the effects of education. Moreover, the protective effect of bilingualism in AD extended to VaD and FTD.

Exploratory analyses were carried out to determine whether speaking three or more languages conferred an added benefit over two languages. No added benefit was found.

## 3. Discussion

The Toronto and Hyderabad studies showed a significant protective effect of speaking two or more languages in delaying onset of AD. In addition, the Hyderabad study extended this finding to FTD and VaD.

In contrast, the Montreal study failed to show an overall protective effect of bilingualism on delaying onset of dementia. However, consistent with the findings of Kavé et al. [34], there was a significant protective effect of speaking four or more languages, as well as a trend in patients who spoke three languages. Analysis of subgroups in the Montreal study showed that bilingualism and multilingualism delayed onset of dementia in immigrants, although there was a surprising finding that native-born Canadian bilinguals developed AD earlier than monolinguals. Also, Canadian born French speaking monolinguals were diagnosed earlier than English speaking monolinguals.

The differences among the studies may reflect the influence of several factors that may offer protection against dementia, including those that relate to culture, socioeconomic status, and linguistic relatedness between languages, demographics, and policies leading to differential status among languages. Differences in oral, literate, and metalinguistic dimensions of language acquisition and use [35], as well as individual and interactional factors [36], may also have impacted the findings. In addition, differences in methodology should be considered. These include methods for establishing age of onset of dementia and definition of immigrant status.

The Toronto and Hyderabad studies defined onset of dementia based on age when symptoms first developed, whereas the Montreal study used age at diagnosis as the main outcome measure in the majority of statistical comparisons. However, a subset of Montreal cases was analysed using age of symptom onset as the outcome measure. There was a protective effect of multilingualism compared to bilingualism or monolingualism in this latter group but there was no effect of bilingualism compared to monolingualism. However, sample size was relatively small with only 54 cases speaking more than one language, a factor that might have led to negative results when comparing bilinguals to monolinguals.

There was a surprising finding in the Montreal study related to immigration status. Whereas bilingualism was protective in immigrants, this effect was not found in native-born Canadians who spoke English. In contrast, immigration status had no effect in the Toronto study. The conflicting findings might at least in part relate to the definition of immigrant versus nonimmigrant status in the Montreal study. As acknowledged by Chertkow et al. [4], there were no direct data on immigrant/nonimmigrant status in the Montreal study. Thus a native-born Canadian was defined as anyone whose first language was native to Canada and those whose first language was not native to Canada were considered to be immigrants. Although Chertkow et al. [4] argued that their estimation of immigrant versus nonimmigrant status was “in the main” correct, the possibility that misclassification of immigrant status may have contributed to the perplexing results in native born Canadians needs to be considered. However, further study is needed.

Although studies from Canada and India suggest a protective effect of bilingualism on dementia, caution must be observed in view of negative findings by others. Zahodne et al. [37] carried out a prospective community-based study of monolinguals and bilinguals from a population of initially nondemented Hispanic immigrants to the United States. Their findings did not support a protective effect of bilingualism on development of dementia, including AD. However, they did not obtain explicit data documenting when subjects began to learn their second language. Sanders et al. [38] examined longitudinal data from the Einstein Aging Study and found that nonnative English speaking status did not have an independent protective effect against developing dementia and AD. Since they did not obtain data on whether native English speakers spoke more than one language, they may have underestimated the degree of bilingualism in their data. In another study, Crane et al. [39] found no protective effect of self-reported use of written or spoken Japanese on development of dementia, including AD.

Although the mechanisms by which bilingualism might delay the onset of dementia are unclear, it has been suggested that this relates to the development of cognitive reserve. Recent neuroimaging data support this concept. Abutalebi et al. [40] examined cerebral grey matter volume in bilingual and monolingual subjects and found more extensive age related decreases in monolingual subjects and significantly increased cerebral grey-matter volume in the left temporal pole in bilinguals. Moreover, there was a significant positive correlation between naming ability in the second language and grey-matter volume in the left temporal pole. In addition, Schweizer et al. [17] used CT scan linear measurements to study bilingual and monolingual patients with AD who were matched for cognitive performance and education. Bilinguals showed significantly greater medial temporal lobe atrophy as compared to monolinguals without any difference in atrophy in other areas. The finding that the bilinguals performed at the same level cognitively as compared to monolinguals, despite greater medial temporal lobe atrophy, supports the concept of greater cognitive reserve in bilinguals. In another study, Luk et al. [18] found evidence for greater white matter integrity in bilinguals compared to monolinguals. These white matter changes might contribute to a neural basis for a protective effect of bilingualism. It has also been observed that individuals who use two or more languages rely on cognitive control mechanisms to inhibit a current task as they engage in activities such as language switching, translation, and language selection [41]. In addition, higher language proficiency (early age of onset/greater duration of exposure) correlates with efficient cognitive control which in turn increases neural activity in prefrontal areas [42].

In conclusion, multicultural studies from Canada and India suggest that there is a protective effect of bilingualism in delaying onset of dementia. In all three studies, the language history of patients had a significant association with onset of dementia but the details of that language history, as well as other contextual factors, mattered and produced different results. Thus, in some contexts bilingualism provided protection, whereas in the context of specific cultural and immigration factors, only multilingualism provided protection. It is clear that bilingualism alone is insufficient to guarantee the postponement of dementia. Future cross-cultural studies are needed to determine the contexts in which bilingualism offers these protective effects and the other factors with which it interacts in order to resolve this important issue.

## Figures and Tables

**Table 1:** Means (and standard deviations) for background measures and age of onset of symptoms of dementia from the two Toronto studies.

Group	*N*	Age at first appointment^a^	Years of education	MMSE^b^	Age at onset^c^
Study 1: [1]					
Monolingual	91	75.4 (9.3)	12.4 (3.8)	21.3 (6.4)	71.4 (9.6)
Bilingual	93	78.6 (8.4)	10.8 (4.2)^1^	20.1 (7.1)	75.5 (8.5)^2^

Study 2: [2]					
Monolingual	109	76.5 (10.0)	12.6 (4.1)	21.5 (5.7)	72.6 (10.0)
Bilingual	102	80.8 (7.7)	10.6^3^ (5.1)	20.4 (5.6)	77.7 (7.9)^4^

^a^Age at first visit to clinic, year.

^b^MMSE: Mini-Mental State Examination (first appointment); maximum score = 30.

^c^Age at which symptoms were first reported by family, year.

^1^ *P* < 0.009, ^2^ *P* < 0.003, ^3^ *P* < 0.003, and ^4^ *P* < 0.0001.

**Table 2:** Age of diagnosis of Alzheimer's disease.

Number of languages spoken	Native English	Native French	Immigrants
1	78.0 (7.0)	72.7 (9.1)	71.4 (8.1)
(*n*)	(289)	(66)	(23)

2	77.9 (7.5)	75.9 (6.5)	76.5 (8.2)
(*n*)	(62)	(24)	(81)

3	79.8 (5.6)	79.5 (2.5)	77.8 (6.4)
(*n*)	(24)	(4)	(39)

≥4	80.7 (3.2)	—	80.9 (5.9)
(*n*)	(3)	—	(15)

Adapted with permission from Lippincott Williams and Wilkins/Wolters Kluwer Health: Alzheimer's Disease and Associated Disorders, [4] 2010. Promotional and commercial use of the material in print, digital, or mobile device format is prohibited without the permission from the publisher Lippincott Williams & Wilkins. Please contact journalpermissions@lww.com for further information.

**Table 3:** Demographic measures and age at onset of symptoms of dementia and its subtypes in Indian study.

Group	*N*	Duration^a^	Years of education	Age at onset of dementia^b^	Age at onset of AD	Age at onset of FTD	Age at onset of VaD
Monolinguals	257	2.1 (1.7)	5.9 (5.1)	61.1 (11.4)	65.4 (10.0)	55.6 (10.5)	57.0 (10.7)

Bilinguals	391	2.3 (1.9)	12.9 (4.9)^1^	65.6 (10.0)^2^	68.6 (9.6)^3^	61.6 (9.0)^4^	60.7 (9.7)^5^

AD: Alzheimer's disease.

FTD: frontotemporal dementia.

VaD: vascular dementia.

^a^Duration of elapsed time between age at onset and age at first appointment.

^b^Age at which symptoms were first reported by family.

^1^ *P* < 0.0001, ^2^ *P* < 0.0001, ^3^ *P* < 0.013, ^4^ *P* < 0.001, and ^5^ *P* < 0.012.
